# The use of daily questions for educational purposes: a TOPday for students

**DOI:** 10.1007/s40037-013-0052-5

**Published:** 2013-04-09

**Authors:** Esther Tanck, Gerjon Hannink, Sascha M. H. F. van Kuppeveld, Sanneke Bolhuis, Jan G. M. Kooloos

**Affiliations:** 1 Orthopaedic Research Laboratory, Radboud University Nijmegen Medical Centre, P.O. Box 9101, 6500 HB Nijmegen, the Netherlands; 2 Department for Evaluation, Quality and Development of Medical Education, Radboud University Nijmegen Medical Centre, P.O. Box 9101, 6500 HB Nijmegen, the Netherlands; 3 Department of Anatomy, Radboud University Nijmegen Medical Centre, P.O. Box 9101, 6500 HB Nijmegen, the Netherlands

## Introduction

Students in biomedical sciences have difficulty understanding biomechanics. In a second-year course, biomechanics is taught in the first week and examined at the end of the fourth week. Knowledge is retained longer if the subject material is repeated [1]. However, how does one encourage students to repeat the subject matter? We investigated if two opportunities to practise per day (TOPday) via email motivated the students to repeat the subject material and to what extent.

## Methods

During the course, all second-year students (n = 92) received a TOPday of biomechanics on every regular course day. The TOPday consisted of one or two multiple-choice questions with increasing difficulty during the course. By clicking an answer, the student received feedback immediately.

At the end of the course, a non-anonymous questionnaire was conducted. Students answered how often they had participated: questions answered none/barely (group A); about half (group B); (almost) all (group C). Other questions on the questionnaire included: level of background knowledge (mathematics/physics), appreciation for a TOPday, and increase (yes/no) of self-confidence and enthusiasm for biomechanics. Test results (scale 1–10) and appreciation versus the number of questions answered were analyzed using one-way ANOVA and Bonferroni post hoc tests. Confidence and enthusiasm were analyzed with descriptive statistics in the group of students who actively participated (groups B and C).

## Results

Eighty-two students participated in the examination and filled in the questionnaire. The appreciation for TOPday in group A (n = 19), B (n = 22) and C (n = 41) was 7.0, 7.4, and 7.8, respectively (*p* < 0.01 between A and C). Of the students, 91 and 79 % showed an increase in their confidence and enthusiasm, respectively, for biomechanics due to TOPday. In addition, students who actively participated (B–C) had a higher test result for biomechanics (*p* < 0.05) compared with those who did not (A). This effect was shown both for students who have difficulties understanding mathematics and physics (n = 20; result A = 3.2, B = 7.1, and C = 7.2) and students who indicated mathematics and/or physics to be easy (n = 62; result A = 5.7, B = 7.2, and C = 7.7).

## Discussion

The teaching method ‘TOPday’ seems an effective way to encourage students to repeat the subject material, with the extra advantage that students are stimulated to keep on practising for the examination. The appreciation was high. With active participation, confidence, enthusiasm, and test results for biomechanics improved. These results will be used to encourage students even more to participate actively in TOPday.

## Conclusion

The students who actively participated in TOPday also had a TOPday during the examination.

## References

[1] Kerfoot BP, DeWolf WC, Masser BA, Church PA, Federman DD (2007). Spaced education improves the retention of clinical knowledge by medical students: a randomized controlled trial. Med Educ.

